# Cerebrovascular Complications and Infective Endocarditis: Impact of Available Evidence on Clinical Outcome

**DOI:** 10.1155/2018/4109358

**Published:** 2018-12-30

**Authors:** Leonardo Schirone, Alessandra Iaccarino, Wael Saade, Mizar D'Abramo, Antonio De Bellis, Giacomo Frati, Sebastiano Sciarretta, Carlos-A. Mestres, Ernesto Greco

**Affiliations:** ^1^Department of Medico-Surgical Sciences and Biotechnologies, Sapienza University of Rome, Latina, Italy; ^2^Department of General and Specialistic Surgery “Paride Stefanini”, Sapienza University of Rome, Italy; ^3^Department of Cardiac Surgery, Humanitas Clinical and Research Center, Rozzano, Milan, Italy; ^4^Department of Cardiovascular, Respiratory, Nephrological, Anesthesiological, and Geriatric Sciences, Sapienza University of Rome, Italy; ^5^Department of Cardiology and Cardiac Surgery, Casa di Cura San Michele, Maddaloni, Caserta, Italy; ^6^IRCCS Neuromed, Pozzilli, Italy; ^7^Department of Cardiovascular Surgery, University Hospital Zurich, Zurich, Switzerland

## Abstract

*Background*. Infective endocarditis (IE) is a life-threatening disease. Its epidemiological profile has substantially changed in recent years although 1-year mortality is still high. Despite advances in medical therapy and surgical technique, there is still uncertainty on the best management and on the timing of surgical intervention. The objective of this review is to produce further insight into the short- and long-term outcomes of patients with IE, with a focus on those presenting cerebrovascular complications.

## 1. Introduction

The term endocarditis refers to an inflammation of the inner tissues of the heart or endocardium that can lead to life-threatening complications if untreated. Although noninfective endocarditis can develop from the deposition of platelet and fibrin thrombi within the four cardiac chambers, the pathology is usually caused by a vegetation colonized by microorganisms, a condition referred to as “infective endocarditis” (IE) [1]. Infective endocarditis is associated with high morbidity and mortality, since the patient may suffer from valvular incompetence leading to heart failure, myocardial abscess, and embolism. The yearly incidence of IE is estimated between 30 and 100 episodes per million persons [2] and is expected to increase in the coming years considering the growing number of interventional procedures, device implantations and other health-related procedures that can provide an access to the bloodstream for the microorganisms. In fact, health-care related procedures are responsible for over one-third of IE in Western countries [2]. Since major predisposing factors are abnormalities in the endocardium including congenital heart defects, rheumatic valvular disease, bicuspid or calcific aortic valves, mitral valve prolapse and hypertrophic cardiomyopathy, a peak of incidence of 145/million persons has been found in the elderly (70-80 years), which are particularly vulnerable also due to reduced efficiency of the immune and hemostatic system [3, 4]. The latter finding may be also partially explained by the increased age of patients undergoing surgical procedures of any kind, mainly with the growth of minimally invasive cardiac procedures [5–8]. Moreover, despite trends toward earlier diagnosis and surgical intervention, the 1-year mortality from IE has not improved in over 2 decades, ranging from 15 to 30% [9]. Around 30% of patients with* Staphylococcu* bacteremia will develop IE at any time. Nowadays prosthetic heart valves, hemodialysis, venous catheters, immunosuppression, pacemakers, and other intracardiac devices and intravenous drug abuse, jointly with the previously mentioned advanced age that often implies comorbidities and frailties of the patient, are considered the main risk factors for the development of IE. In detail, almost 75% of IE patients have structural valve abnormalities (e.g., mitral valve prolapse, bicuspid aortic valve) or have implanted prosthetic valve [10].

**Figure 1:**
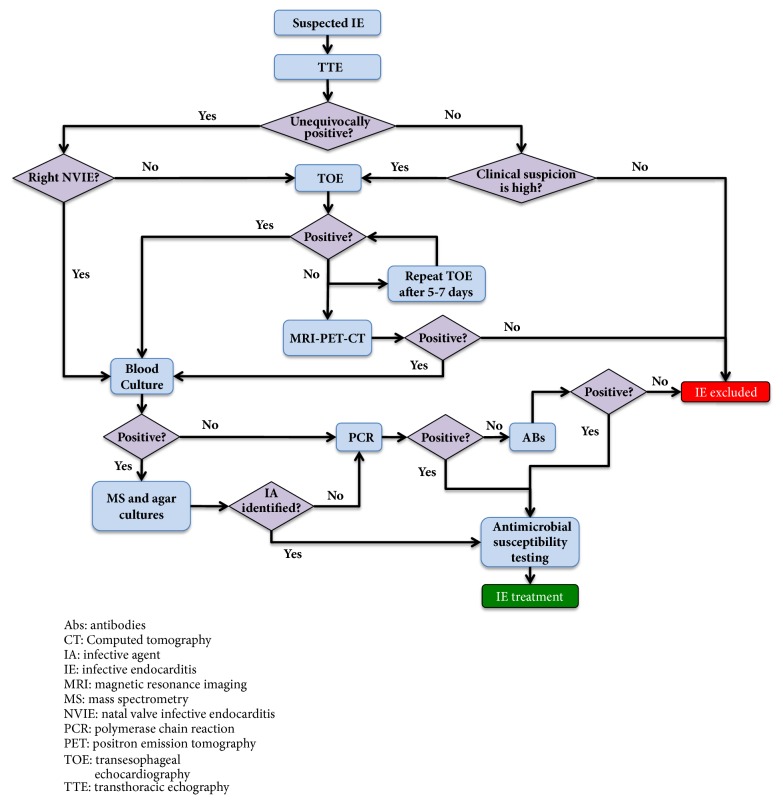
A schematic representation of the diagnostic algorithm according to the 2015 ESC guidelines.

IE was traditionally assessed following the “Duke criteria”, but recently these criteria have been updated to the state of the art by 2015 ESC guidelines [11]. Briefly, when IE is suspected, transthoracic echocardiography (TTE) should be performed first, followed by transesophageal echocardiography (TOE) in those cases of uncertainty or negative results in which the clinician highly suspects the presence of bacterial vegetation. Magnetic resonance imaging (MRI), computed tomography (CT) and positron emission tomography-computed tomography (PET-CT) may be helpful to detect microbial vegetation in difficult echocardiographic settings and, importantly, may detect symptomatic or asymptomatic distal site of embolization. The identification of the microbial agent from blood samples is the necessary following step. If blood cultures are positive, then mass spectrometry and agar culturing should be performed to identify the infective agent; if blood cultures are negative, serological analyses by PCR should be performed. If those are still negative, the use of antinuclear, antiphospholipid, and anti-Pork antibodies is then recommended [11]. Antimicrobial susceptibility testing concludes this diagnostic algorithm and allows the clinician to administrate an effective antibiotic therapy to the patient (Figure 1).

Surgery should be considered as part of the therapy for IE as it is required in 25% to 50% of cases in the acute phase [12, 13]. Surgery is more frequently indicated on prosthetic valve endocarditis (PVE) than in native valve endocarditis (NVIE). Selecting for urgent surgery those patients that are exposed to the highest risk of death before decompensation dramatically ameliorates the course of the disease and improves prognosis. In fact, patient comorbidities and preoperative status, together with the specific infecting microorganism and intracardiac anatomy, are crucial for the surgical outcome, as confirmed by preoperative echocardiogram and intraoperative inspection. The major risk factors for death are heart failure (HF), periannular complications, and/or* S. aureus* infection [14]. This latter pathogen, compared to other bacteria, is more frequently associated with neurological complications. Clinically relevant neurological complications are present in up to 30% of the cases but may occur in 35-60% as silent embolization that may lead to cerebral or subarachnoidal hemorrhage, silent ischemic attacks, meningitis, toxic encephalopathy, and eventually brain abscess. Practice guidelines have been recently elaborated to assess an appropriate timing for surgery (urgent versus emergent), to avoid progressive HF, irreversible structural damage, and to prevent systemic embolism (Table 1) [11].

The aim of this review is to focus on the decision-making process in the setting of IE and concomitant neurological events like ischemic stroke or intracranial hemorrhage. The management of these patients is difficult and complex and requires a multidisciplinary approach (Endocarditis Team).

## 2. Infective Endocarditis and Neurological Complications

Intracardiac vegetation may result in life-threatening embolic events. The overall risk of peripheral embolism is 20-50%, and once antibiotic therapy is started, this risk decreases to 6-21% [15]. The brain and the spleen are the most frequent sites of embolization in left-sided IE, while pulmonary embolism is frequent in native right-sided and pacemaker lead IE. The overall incidence of embolic stroke during IE ranges from 10 to 50% and its associated mortality is over 30%. However, embolic events may be silent in up to 50% of the patients, in particular those affecting the spleen and the brain. In this large amount of cases, the diagnosis relies mainly on noninvasive imaging, among which echocardiography has a prominent role, allowing to determine size, location, and evolution of the vegetation also in response to antibiotic therapy in a fast and economical way. The risk of embolization is related to specific microorganisms as* S. aureus*,* S. bovis,* or Candida spp., previous embolism, multivalvular IE, and biological markers [15–17]. The size and mobility of the vegetation are powerful independent predictors of a new embolic event [18] and a recent study found that the risk of neurological complications was particularly high in patients with large (> 30 mm length) vegetation [19]. Vegetation located on the anterior leaflet of the mitral valve may embolize more often. Neurological manifestations can occur both before or after the diagnosis of IE is made and recurrent events can develop also later during the infection. Clinical presentation is variable and includes stroke, transient ischemic attack, intracerebral or subarachnoid hemorrhage, brain abscess, meningitis, and toxic encephalopathy. Focal signs predominate and evidence supports that clinically silent cerebral embolisms occur in 35–60% of IE patients [20].

## 3. Timing of Surgery

Evidence regarding the optimal time interval between stroke and cardiac surgery is conflicting. During the acute phase of ischemic stroke, the occurrence of hypotension and the need for full anticoagulation during cardiopulmonary bypass may worsen the neurological status, due to an augmentated risk of hemorrhagic transformation and a potential extension of the ischemic lesion [21]. However, several studies suggested that the risk of postoperative neurological deterioration after early surgery is lower than previously expected even in patients with cerebral hemorrhage. After an ischemic stroke, cardiac surgery is not contraindicated unless the neurological prognosis is judged poor [22]. In the study from the ICE-PCS collaboration, the outcome of 58 patients with an ischemic stroke undergoing early surgery (<7days) was compared with late surgery. After risk adjustment, surgery was associated with a nonsignificant increase of in-hospital mortality [23]. This finding has been interpreted by both the American Heart Association (AHA) and the European Society of Cardiology (ESC) to suggest that surgery can be safely performed if indicated, although stroke remains a common reason for denial or delaying surgical intervention in everyday practice. In contrast, transient ischemic attack or silent embolism should not delay surgery that is indicated for other reasons. If cerebral hemorrhage has been excluded by cerebral computed tomography and neurological damage is not severe (i.e., coma), surgery could be performed with low neurological risk (mortality 3–6%) and chances of complete neurological recovery [24].

On the contrary, in cases of hemorrhagic lesions, recent European guidelines recommend delaying surgery for at least 1 month. A recent Japanese multicenter study [25] shows that delayed surgery (2 weeks after cerebral infarction) results in a higher incidence of in-hospital death in the group of patients affected by nonhemorrhagic cerebral infarction. Patients who had surgery between 15 and 28 days or after 29 days from the onset of the embolic event had higher incidences of in-hospital death compared with those who had surgery within 7 days. On the other hand, in the group of patients affected by hemorrhagic cerebral infarction, those who had surgery between 8 and 21 days or after 22 days after the onset had a lower incidence of in-hospital death compared with those who had surgery within 7 days. Even if these differences were not statistically significant, early surgery appears safe but very early surgery (before 7 days) should be avoided in patients with hemorrhagic infarction. However, the evidence is still low with this regard.

## 4. Anticoagulant Therapy

The treatment of stroke patients with anticoagulant therapy may lead to hemorrhagic transformation in 51-71% of the cases, considerably worsening the severity of the neurologic symptoms and the prognosis [26, 27]. However, the chance of developing this condition is also depending on the size, location, and cause of the stroke [28]. The management of these patients depends on the amount of bleeding and may require clot evacuation for the most severe cases. An eventual fatal outcome is typically due to brain hemorrhage and central nervous system complications. Normally, thrombus organization requires two weeks and the administration of anticoagulant agents should be well pondered, strictly monitoring prothrombin-time (PT) during the gradual reintroduction of the therapy [29]. Interestingly, the therapeutic safety and benefits of anticoagulation in patients with native valve infective endocarditis (NVIE) that are suffering from ischemic stroke are still debated [30].

Prosthetic valve infective endocarditis (PVE) occurs at a rate of about 2% per year, and mortality ranges from 50 to 80%. The management of mechanical valve PVE, whether medical or surgical, remains a challenging area as outcomes are frequently dismal. One of the salient issues in the medical management of mechanical valve PVE is the use of anticoagulant therapy, which is sometimes recommended to prevent atherothrombotic complications in case of mechanical valve prosthesis or presence of atrial fibrillation. However, it is recommended to avoid all form of anticoagulation in those patients suffering from PVE caused by* S. *aureus that have recently been affected by a neuroembolic event. In fact,* S. aureus *PVE is far more lethal than the forms caused by other infective agents, and anticoagulation should be avoided during the first two weeks of antibiotic therapy, and an eventual reintroduction carefully considered [31]. Some authors argue that the use of anticoagulants in the setting of an infected mechanical prosthetic valve increases the potential risk of secondary cerebral hemorrhage in the event of an embolic stroke. In contrast, others affirm that anticoagulant therapy should not be discontinued in mechanical valve PVE because of the increased thromboembolic risk. Patients with a large infarcted area, uncontrolled hypertension, evidence of coagulopathy, or possible hemorrhagic transformation should be considered for discontinuation of anticoagulant therapy, since in some cases the benefits may be outweighed by the risk of hemorrhage.

## 5. Conclusions

Preoperative neurological events represent a conflicting situation for the treating team. Patients with neurological events frequently present with heart failure, uncontrolled infection, and the risk of further embolization if large vegetation is present. Furthermore, there is a risk of progression of neurological damage if patients are on antithrombotic therapy or undergo cardiac surgery as full anticoagulation is mandatory for cardiopulmonary bypass. In this conflicting setting, the timing for surgery is still a matter of concern and there still exists uncertainty with regard to the right timing for surgery. Well-designed and adequately powered randomized studies are difficult to organize due to a number of factors including ethical issues. Concluding remarks include the following:

(i) The emphasis on “early surgery” differs mainly between European and US practice guidelines. The ESC guidelines distinguish emergency surgery (performed within 24 h), urgent surgery (within a few days), and elective surgery (after 1 to 2 weeks of antibiotic therapy), with surgery advised on an urgent basis for most of the cases. In contrast, the AHA guidelines define early surgery as “during initial hospitalization and before completion of a full course of antibiotics.”

(ii) In the case of ischemic stroke, early surgery, even within 72 hours, may improve prognosis without increasing the rate of hemorrhage conversion [32].

(iii) If required because of hemodynamic instability, in case of hemorrhagic lesions, surgery may be performed after careful evaluation of sizes and etiologies by a multidisciplinary discussion within 4 weeks of the hemorrhagic event if patients are neurologically viable. In a number of studies, the preoperative time interval between the neurological event and surgery was found to be shorter in nonsurvivors, but a short preoperative time tends to be associated with severe hemodynamic impairment and ongoing sepsis, which are associated with higher mortality. The plan of action for patients with minor bleeding or minor hemorrhagic conversion of an ischemic stroke remains open to clinical judgment.

(iv) The eventual discontinuation of anticoagulant therapy should be considered carefully for every single case, evaluating whether the risk of hemorrhage outweighs the benefit of continuing the therapy.

## Figures and Tables

**Table 1:** Indications and timing of surgery in left-sided valve IE (native valve endocarditis and prosthetic valve endocarditis) ESC-EACTS 2015 Guidelines [11].

**Heart failure**	**Surgical Timing**
Aortic or mitral NVE or PVE with severe acute regurgitation, obstruction or fistula causing refractory pulmonary oedema or cardiogenic shock	**Emergency** (Class I - Level of recommendation B)

Aortic or mitral NVE or PVE with severe regurgitation or obstruction causing symptoms of HF or echocardiographic signs of poor haemodynamic tolerance	**Urgent** (Class I - Level of recommendation B)

**Uncontrolled infection**	

Locally uncontrolled infection (abscess, false aneurysm, fistula, enlarging vegetation)	**Urgent** (Class I - Level of recommendation B)

Infection caused by fungi or multiresistant organisms	**Urgent/Elective** (Class I - Level of recommendation C)

Persisting positive blood cultures despite appropriate antibiotic therapy and adequate control of septic metastatic foci	**Urgent** (Class II - Level of recommendation A)

PVE caused by staphylococci or non-HACEK gram-negative bacteria	**Urgent/Elective ** (Class II - Level of recommendation A)

**Prevention of embolism**	

Aortic or mitral NVE or PVE with persistent vegetation >10 mm after one or more embolic episode despite appropriate antibiotic therapy	**Urgent** (Class I - Level of recommendation B)

Aortic or mitral NVE with vegetation >10 mm, associated with severe valve stenosis or regurgitation, and low operative risk	**Urgent ** (Class II - Level of recommendation A)

Aortic or mitral NVE or PVE with isolated very large vegetation (> 30 mm)	**Urgent** (Class II - Level of recommendation A)

Aortic or mitral NVE or PVE with isolated large vegetation (>15 mm) and no other indication for surgery	**Urgent** (Class II - Level of recommendation B)
